# Deletion of PTEN produces autism-like behavioral deficits and alterations in synaptic proteins

**DOI:** 10.3389/fnmol.2014.00027

**Published:** 2014-04-16

**Authors:** Joaquin N. Lugo, Gregory D. Smith, Erin P. Arbuckle, Jessika White, Andrew J. Holley, Crina M. Floruta, Nowrin Ahmed, Maribel C. Gomez, Obi Okonkwo

**Affiliations:** ^1^Department of Psychology and Neuroscience, Baylor UniversityWaco, TX, USA; ^2^Institute of Biomedical Studies, Baylor UniversityWaco, TX, USA

**Keywords:** Pten, PI3K/AKT/mTOR, FMRP, Kv4.2, autism spectrum disorders, autism, repetitive behavior, mGluR

## Abstract

Many genes have been implicated in the underlying cause of autism but each gene accounts for only a small fraction of those diagnosed with autism. There is increasing evidence that activity-dependent changes in neuronal signaling could act as a convergent mechanism for many of the changes in synaptic proteins. One candidate signaling pathway that may have a critical role in autism is the PI3K/AKT/mTOR pathway. A major regulator of this pathway is the negative repressor phosphatase and tensin homolog (PTEN). In the current study we examined the behavioral and molecular consequences in mice with neuron subset-specific deletion of PTEN. The knockout (KO) mice showed deficits in social chamber and social partition test. KO mice demonstrated alterations in repetitive behavior, as measured in the marble burying test and hole-board test. They showed no changes in ultrasonic vocalizations emitted on postnatal day 10 or 12 compared to wildtype (WT) mice. They exhibited less anxiety in the elevated-plus maze test and were more active in the open field test compared to WT mice. In addition to the behavioral alterations, KO mice had elevation of phosphorylated AKT, phosphorylated S6, and an increase in S6K. KO mice had a decrease in mGluR but an increase in total and phosphorylated fragile X mental retardation protein. The disruptions in intracellular signaling may be why the KO mice had a decrease in the dendritic potassium channel Kv4.2 and a decrease in the synaptic scaffolding proteins PSD-95 and SAP102. These findings demonstrate that deletion of PTEN results in long-term alterations in social behavior, repetitive behavior, activity, and anxiety. In addition, deletion of PTEN significantly alters mGluR signaling and many synaptic proteins in the hippocampus. Our data demonstrates that deletion of PTEN can result in many of the behavioral features of autism and may provide insights into the regulation of intracellular signaling on synaptic proteins.

## INTRODUCTION

In the last few years the rate of children with autism spectrum disorders (ASDs) has risen from a prevalence rate of 0.6 % in 2000 to the current rate of 1 in 88, as reported by the Centers for Disease Control and Prevention (CDC; 2012). Several genetic studies have identified a large number of genes that underlie ASDs (Abrahams and Geschwind, 2008). Many of the genes implicated in ASDs encode synaptic proteins such as the scaffolding proteins Shank 3, Homer, and PSD-95, and the synaptic adhesion molecules neuroligin and neuroexin (Delorme et al., 2013). However, many of these gene mutations are rare and only account for a small fraction of the cases of ASDs. Another approach to understanding the molecular mechanisms underlying ASDs has been to examine single gene mutations that share a high comorbidity with ASD such as fragile X syndrome, Tuberous sclerosis complex, Timothy Syndrome, Rett Syndrome, and Angelmen syndrome (Matsuura et al., 1997; Baker et al., 1998; Amir et al., 1999; Splawski et al., 2004; Hatton et al., 2006). The research in rare genetic disorders and in neurological syndromes that share high comorbidity with ASD has revealed that an underlying disruption in cell signaling pathways could act as a common convergent pathway for many of these disorders (Ebert and Greenberg, 2013). There is a growing consensus that gene mutations associated with the regulation of the phosphoinositide 3-kinase/AKT/mammalian target of rapamycin (PI3K/AKT/mTOR) intracellular signaling pathway play a significant role in mediating the behavioral abnormalities that characterize autism.

Alterations in the PI3K/AKT/mTOR pathway results in many behavioral abnormalities and is expected to play a significant role in ASD. Alteration of the mTOR signaling pathway has been shown to be involved in 14% of ASD individuals (Kelleher and Bear, 2008; de Vries, 2010). In particular the phosphatase and tensin homolog on chromosome 10 (PTEN) gene may be a significant regulator of this pathway in mediating the ASD phenotype. In a clinical cohort of pediatric patients with ASD there is a prevalence rate of 8.3% with mutations in PTEN (Varga et al., 2009). Additionally subjects with developmental delay/mental retardation have a higher prevalence rate of 12.2% with a mutation in PTEN (Varga et al., 2009).

Since deletion of the PTEN gene is embryonically lethal, several conditional knockouts (KO) of PTEN have been created (Kazdoba et al., 2012; Sperow et al., 2012). The neuronal specific KO of PTEN (NS-Pten KO) that will be used in this experiment results in deletion of PTEN in a subset of postmitotic neurons in the cortex, hippocampus, and cerebellum (Backman et al., 2001; Kwon et al., 2001). A different cre conditional Pten KO has been shown to have social behavior deficits and deficits in learning and memory (Kwon et al., 2006). However, the conditional KO used in the current study has not been investigated for social behavior deficits. In addition, repetitive behaviors and vocalizations are two other features of autism that have not been investigated in mice with deletion of PTEN. One of the goals of this study is to more comprehensively examine the behavioral alterations due to deletion of PTEN in the brain.

Previous work has also found that absence of PTEN dramatically increases dendrite size and results in an enlarged hippocampus (Backman et al., 2001; Kwon et al., 2001; Meikle et al., 2008). Deletion of PTEN also results in aberrant LTP and LTD, but the molecular mechanism underlying these changes is not known (Wang et al., 2006; Sperow et al., 2012; Takeuchi et al., 2013). Therefore, another objective of the experiments in this paper will be to determine the synaptic proteins that are altered in the hippocampus due to deletion of PTEN.

## MATERIALS AND METHODS

### ANIMALS

Neuron subset-specific Pten conditional mice have been previously described as GFAP-Cre; Pten^loxP/loxP^ (Backman et al., 2001; Kwon et al., 2001). They are on a FVB-based mixed background strain and have been bred for more than 10 generations. We bred NS-Pten^loxP/^^+^ heterozygote parents to produce NS-Pten^+^^/^^+^ wildtype (WT), NS-Pten^loxP/^^+^ heterozygous (HT), and Pten^loxP/loxP^ KO. These mice were generated and housed at Baylor University at an ambient temperature of 22°C, with a 14-h light and 10-h dark (20:00 to 6:00 h) diurnal cycle. The mice were given *ad libitum* access to food and water. All procedures conducted with mice were in compliance with the National Institutes of Health Guidelines for the Care and Use of Laboratory Animals and the animal protocol was approved by Baylor University Animal Care and Use Committee. For the behavioral tests we used mice on postnatal days 10 and 12 to examine ultrasonic vocalizations (UV). This time was used since mice produce few vocalizations after this time period. For the reminder of the behavioral tests we used mice from the age of 6 to 9 weeks. We used this narrow age of testing to reduce the variability of testing at different ages. In addition, these tests have all been previously conducted during this age period.

### SOCIAL BEHAVIOR

#### Three chamber social behavior test

Mice were placed in a clear acrylic box with three chambers as previously described (Nadler et al., 2004). Mice were tested in two conditions. In the first, a mouse was placed in the center chamber then allowed to freely explore the chamber. The corners of the two side chambers housed empty black wire-mesh cylinders. Containers were placed on top of the cylinders to prevent the mice from climbing above the wire mesh cylinders. After the 10 min trial it was placed in the center area of the chamber and the entry to the side chamber was obstructed. In the second condition, an unfamiliar mouse (gender-, age-, and weight-matched) was placed in one cylinder and a similar sized black block object was placed in the other cylinder. The placement of the novel mouse and object were counterbalanced across trials to prevent bias in response. The barriers to the side chambers were then removed and the time and frequency in the three chambers and at the cylinders was recorded for the 10 min testing period.

#### Social partition

A partition test evaluated social behaviors in mice during a non-contact version of a social interaction test (Spencer et al., 2005). The experimental animals were individually housed for 24 h on one side of a standard cage that was divided in half by a clear perforated (0.6 cm-diameter holes) partition. The other side of the partition housed a partner mouse (gender-, age-, and weight-matched). Each side had individual water feeding tubes (Bioserv 9019 50 ml tubes) for water consumption and were given food pellets overnight. The day of testing consisted of three 5 min testing periods. The first test was with a familiar mouse and then testing occurred with an unfamiliar animal. The third test was with the reintroduction of the familiar mouse. We measured the total time at the partition and the frequency of visits at the partition of the experimental mouse.

#### Olfactory habituation/dishabituation test

We evaluated the ability of the mouse to detect novel odors and social odors using a test previously described (Yang and Crawley, 2009). In this test of olfactory function we first transported the experimental mouse to a holding room for 45 min. After this acclimation period we individually tested each animal in a clean mouse cage. We dipped a cotton swab in a test tube with different solutions. The solutions were water, almond, banana, and social odor 1, and social odor 2. We presented each odor for 2 min and measured the time the animal spent at the cotton tip for each 2 min trial. Each odor was presented three times. The social odors were created by swabbing the cotton tip in a zigzag fashion in previously soiled bedding from mice the experimental animal had not interacted with. This was performed for two separate cages to create social odor 1 and 2. After testing the animal was returned to its home cage.

### REPETITIVE BEHAVIOR AND ULTRASONIC COMMUNICATION

#### Hole-board test

The hole-board and marble tests have been used to examine the repetitive behavior (stereotypy) aspect of autism (Hoeffer et al., 2008; Jamain et al., 2008). The mice were placed into a clear acrylic arena (40 cm × 40 cm × 30 cm) that had hole-board inserts. The boards had 16 equidistant floor holes, 3 cm in diameter. Hole poke activity was scored by an individual blind to the condition of the mice. We measured the total number of holes poked, latency to first hole poke, number in center, and number of repeated pokes in the same hole during the 10 min test.

#### Marble burying

This task measures the tendency of mice to dig and bury objects. Mice were placed in a new home cage that had approximately 3 cm of bedding. 20 marbles were placed equidistant from each other in a grid pattern. We measured the number of marbles buried at the 75% level and number of marbles completely buried in a 30 min test.

#### Ultrasonic vocalizations

To examine the communication aspect of autism in animal models we examined isolation-induced UV in pups (Jamain et al., 2008; Scattoni et al., 2008). One cohort of animals was tested on postnatal day 10 and another cohort of animals was tested on postnatal day 12. The mice were individually placed in a chamber that has four UV detectors set to 40, 50, 60, and 70 kHz. Young mice will emit UVs when removed from their mother. We measured the number of vocalizations the mice emitted in a 5 min test using the automated detection software (Ultravox software by Noldus, Netherlands). The mice were then placed in a housing pan with clean bedding after testing. The cage was kept warm by a heating pad under the cage. The pups were then returned to the home cage after all mice were tested. No more than five pups were tested in any given litter so the time separated from the mother was 30 min or less.

### LOCOMOTOR ACTIVITY AND ANXIETY

#### Open field

Locomotor activity was evaluated through the use of the open field activity test as previously described (Lugo et al., 2012). The mice were placed into a clear acrylic arena (40 cm × 40 cm × 30 cm) to investigate their activity levels for 30 min. Activity in the open field was collected by a computer-operated optical animal activity system (Fusion by Omnitech Electronics, Inc., Canada, USA). We measured total activity levels, stereotypy (self-grooming), rearing, and circling behavior. We also measured the time and distance the animal spent in the center and in the perimeter of the open field to determine differences in anxiety.

#### Elevated Plus-maze

The elevated plus maze was used to examine alterations in anxiety. The elevated-plus maze apparatus consists of two enclosed and two open horizontal perpendicular arms (30 cm × 5 cm) positioned 40 cm above the floor. There is a central square platform (5 cm × 5 cm) that forms from the connection of the four arms. We monitored the mouse movement in the elevated-plus maze by video tracking software (Noldus: Ethovision; Netherlands) and recorded the videos using a video capturing device (Dazzle^®^ video creator plus HD, Corel, Canada). We measured the time and frequency of the mice in the open arms, center arms, and closed arms in a 10 min test. An increase in time spent in open arms compared to closed arms is generally believed to be an index of lowered anxiety. We also measured headdips in the open arms and rearing in the closed arms when we examined the video files at a later time.

### WESTERN BLOTTING

Mice were sacrificed at approximately 8 weeks of age. Hippocampi were rapidly dissected, rinsed in 1X phosphate buffer solution, and placed on dry ice. All samples were then stored at -80°C until used. Hippocampi were homogenized in ice-cold homogenization buffer (0.32 M sucrose, 1 mM EDTA, 5 mM Hepes) containing protease inhibitor cocktail (Sigma, USA) and processed for western blotting as previously described (Lugo et al., 2008). Through this procedure we produced total homogenate samples and crude synaptosomes. The total homogenate samples were used for total and phosphorylated S6, total and phosphorylated AKT, and S6K. The crude synaptosomes were used for FMRP, phosphorylated FMRP, group 1 metabotrophic glutamate receptors (mGlurR), Kv4.2, and all synaptic proteins. The protein concentration was determined using the Bradford Protein Assay (Bio Rad, Hercules, CA, USA). The samples were normalized to a common protein amount and diluted in Laemmli loading buffer (4X: 0.25 M Tris, pH 6.8, 6% SDS, 40% Sucrose, 0.04% Bromophenol Blue, 200 mM Dithiothreitol). Following SDS-PAGE, proteins were transferred to Hybond-P polyvinyl difluoride membranes (GE Healthcare, Piscataway, NJ, USA). Membranes were incubated in blocking solution [5% non-fat milk diluted in 1X Tris Buffered Saline (50 mM Tris-HCl, pH 7.4, 150 mM NaCl) with 0.1% Tween (1X TBS-T) and 1 mM Na_3_VO_4_] for 1 h at room temperature (RT). Membranes were then incubated overnight at 4°C with the primary antibodies diluted in blocking solution. The primary antibodies used were as follows: ribosomal S6 protein, P-S6 (S235/236), AKT, P-AKT (S473), S6K, FMRP (1:2K; Cell Signaling Technology, Boston, MA, USA); Kv4.2, PSD-95, Sapap1, sap102, pan-shank, SAP97, CASK, Ankyrin-B, Neuroligin-1, Neuroligin-3, mortalin (1:1K; NeuroMab, Davis, CA, USA); pFMRP (Cambridge, UK), and actin (1:5K; Sigma Chemical Co., USA). Following incubation in primary antibodies membranes were washed in 1X TBS-T (3 × 5 min). Membranes were then incubated with horseradish peroxidase labeled secondary antibodies: goat anti-rabbit IgG or anti-mouse IgG (1:10K; Cell Signaling Technology, Boston, MA, USA). After washes in 1X TBS-T membranes were incubated with GE ECL Prime (GE Healthcare, Piscataway, NJ, USA) and immunoreactive bands were captured by a western blot imaging system (ProteinSimple, Santa Clara, CA, USA).

Optical density of immunoreactive bands was measured using the ProteinSimple AlphaView software. Optical densities obtained for all bands from phospho-proteins were normalized to the levels of the total protein from the same sample, and expressed as ratio of phospho to-total protein. Optical densities obtained for all bands of interest were normalized for loading to the actin or mortalin levels within the same lane. Each experimental point represents a single mouse (*n* = 1). All groups were normalized to the average of the control group (Pten WT). The values for the WTs represent biological replicates and were collected from littermate WT mice.

## RESULTS

### PTEN DELETION RESULTS IN SOCIAL BEHAVIOR DEFICITS AND DOES NOT ALTER NOVEL ODOR DISCRIMINATION

**FIGURE 1:**
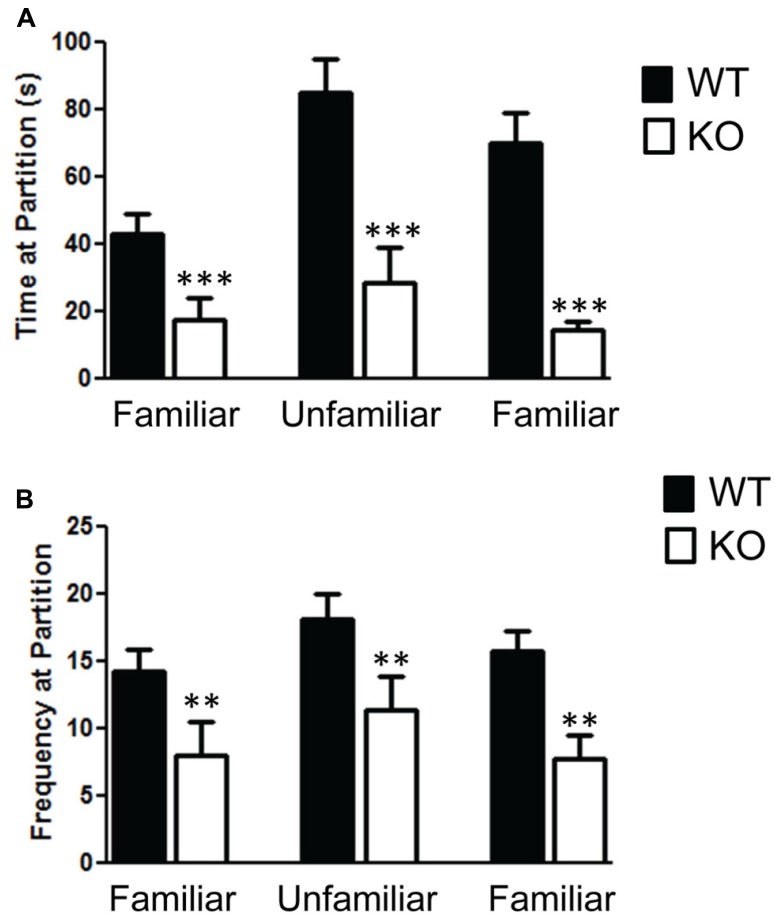
**The Pten mutant mice show deficits in the social partition test. (A)** NS-Pten knockout (KO) mice show a decrease in time spent at the partition in the social partition test. **(B)** Similar results were found in the frequency of visits to the partition across the three conditions. The bars represent the mean with Standard Error of the Mean. The asterisks (*** or **) indicate a significant group difference at respective *p* values (*p* < 0.001 or *p* < 0.01, respectively).

The social partition and social chamber tests both reveal a significant impairment in social behavior in the NS-Pten KO mice. We found a significant impairment in the social partition test in NS-Pten KO mice. A two-way ANOVA shows a significant main effect of group *F*(1,23) = 23.1, *p* < 0.001 across the three trials in the partition test (**Figure 1A**), *n* = 11, 10 for WT and KO, respectively. The NS-Pten KO mice showed significant suppression of social interaction at the partition with familiar and unfamiliar mice. Similar deficits in social behavior were found in the frequency at the partition between the groups *F*(1,23) = 10.8, *p* < 0.01 (**Figure 1B**).

**FIGURE 2:**
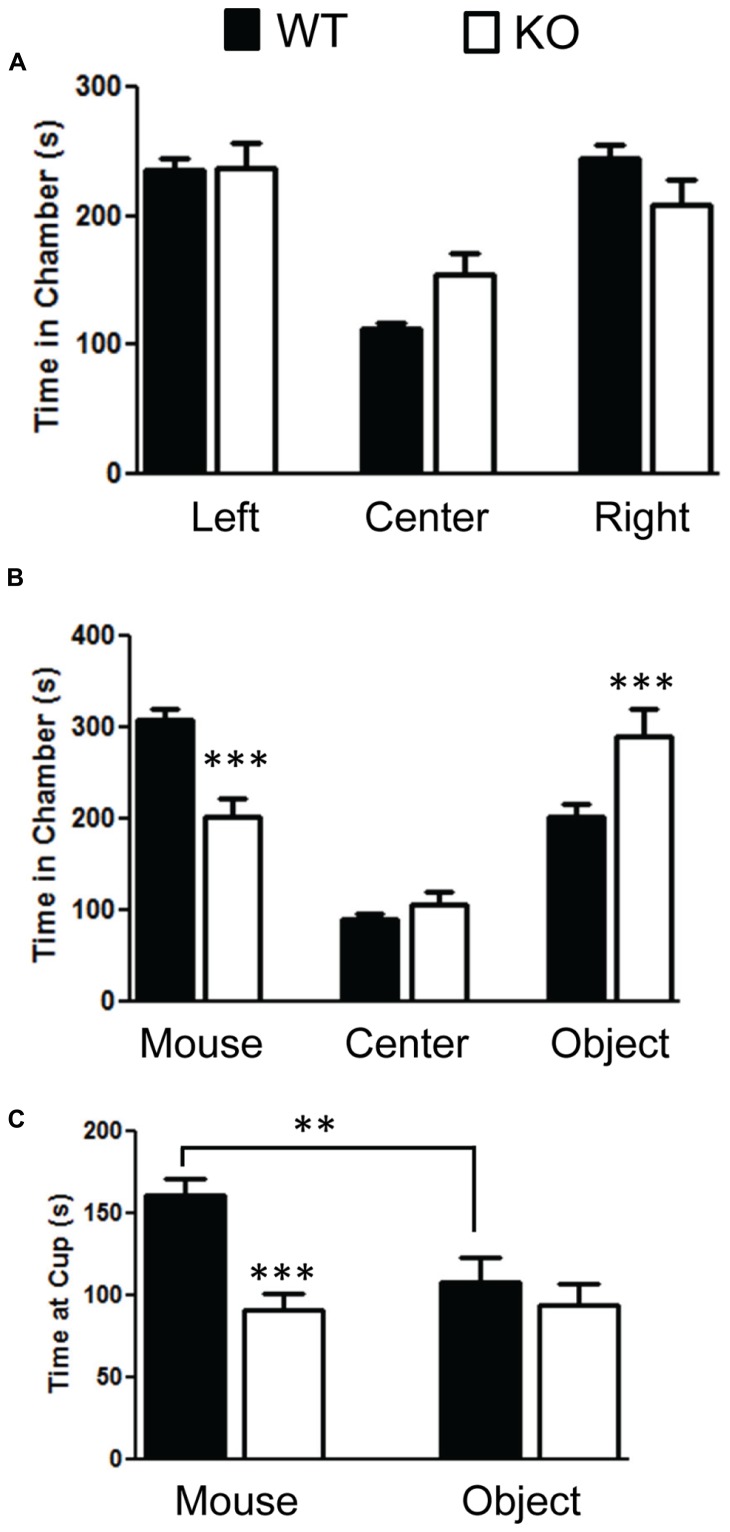
**Mutation in PTEN results in social behavior deficits in the three chamber social behavior test. (A)** Wildtype (WT) and NS-Pten (KO) mice spent equal time across chambers in the habituation phase. **(B)** KO mice spent less time in the chamber that housed the mouse than the WT, spent equal amount of time compared to WT in center, and spent more time than WT in the chamber that housed the novel object. **(C)** The KO mice spent less time at the cup that housed the mouse than WT mice. The bars represent the mean with Standard Error of the Mean. The asterisks (*** or **) indicate a significant group difference at respective *p* values (*p* < 0.001 or *p* < 0.01, respectively).

We found significant impairment of social behavior in the NS-Pten KO in the three chamber test. We did not find any bias in side preference in phase A of the three chamber social test (**Figure 2A**). A repeated-measures test found no difference between the groups *F*(1,28) = 1.9, *p* = 0.18. There was a main effect of time in each chamber *F*(2,56) = 20.0, *p* < 0.001 but no group × chamber interaction *F*(2,56) = 2.37, *p* = 0.1. The sample size was 31 for WT and 20 KO. In phase B where a mouse was placed in a cup in one of the chambers and a novel object was placed in the cup in the other chamber there was evidence of social behavior deficits in the NS-Pten KO mice. The NS-Pten KO mice show less time in the chamber with the mouse, more time in the chamber with the novel object, and spend less time at the cup with a mouse compared to WT mice (**Figure 2B**). There was not a main effect of group *F*(1,27) = 0.83, *p* = 0.77, but there was a main effect of chamber condition *F*(2,54) = 36.0, *p* <0.001 and there was an interaction between group and chamber *F*(2,54) = 11.2, *p* < 0.001. We then used separate t-tests to compare differences between the groups in each chamber and found significant statistical difference in the time spent in the chamber with the mouse *t*(1,27) = 4.7, *p* < 0.001 and in the chamber that housed the novel object *t*(1,27) = 2.7, *p* < 0.05, but no difference in time in the center chamber *t*(1,27) = 1.2, *p* = 0.25. We then analyzed the time the animal spent at the cups in the chamber. We found that the NS-Pten KO mice spent less time at the cup that housed the mouse compared to the WT mice *t*(1,25) = 4.7, *p* < 0.001 and no difference in time spent at the novel object compared to the WT mice *t*(1,25) = 0.6, *p* = 0.5 (**Figure 2C**). Furthermore, the WT mice show a significant increase in time spent at the cup that housed the mouse than the cup with the novel object when analyzed with a paired *t*-test *t*(1,14) = 3.2, *p* < 0.01. The NS-Pten KO mice did not show a difference across the cups when using a paired *t*-test *t*(1,11) = 0.18, *p* = 0.86.

**FIGURE 3:**
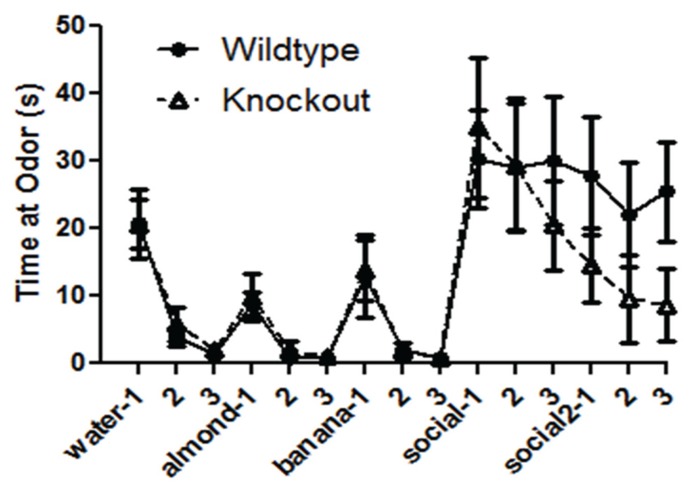
**Deletion of PTEN does not alter the ability of the mice to habituate and dishabituate to an odor.** Wildtype (WT) and knockout (KO) mice had similar patterns of habituation and dishabituation to novel odor and social odors repeatedly presented. The bars represent the mean with Standard Error of the Mean.

The WT and NS-Pten KO mice demonstrated the ability to detect different novel odors, habituate to them, and demonstrated dishabituation to the novel odors when repeatedly presented (**Figure 3**). In each presentation there was a significant decrease in time spent at the odor across the three trials. There was no difference between groups for time spent sniffing the cotton tip with water *F*(1,30) = 0.24, *p* = 0.62, but there was a significant change over the three trials *F*(2,60) = 35.5, *p* < 0.001. There was no difference between groups for time spent sniffing the cotton tip with almond *F*(1,30) = 0.38, *p* = 0.54, but there was a significant change over the three trials *F*(2,60) = 20.5, *p* < 0.001. There was no difference between groups for time spent sniffing the cotton tip with banana *F*(1,30) = 0.05, *p* = 0.82, but there was a significant change over the three trials *F*(2,60) = 11.4, *p* < 0.02. There was no difference between groups for time spent sniffing the cotton tip with the first social odor *F*(1,30) = 0.01, *p* = 0.98, and there was no change over the three trials *F*(2,60) = 0.72, *p* = 0.49. There was a marginal difference in the time spent sniffing the cotton tip with the second social odor *F*(1,30) = 3.0, *p* = 0.09. There was no change across the three trials *F*(2,60) = 0.51, *p* = 0.60. The results from the social odors suggest that the NS-Pten KO and WT mice did not habituate to the social odors. The sample size was 17 for WT and 16 for KO. However, they responded similarly to the presentation of social odor 1 and to social odor 2. These results suggest that the NS-Pten KO mice have a functional olfactory discrimination system and can habituate to odors when presented multiple times.

## PTEN DELETION RESULTS IN REPETITIVE BEHAVIOR DEFICITS BUT DOES NOT IMPACT ULTRASONIC VOCALIZATIONS

**FIGURE 4:**
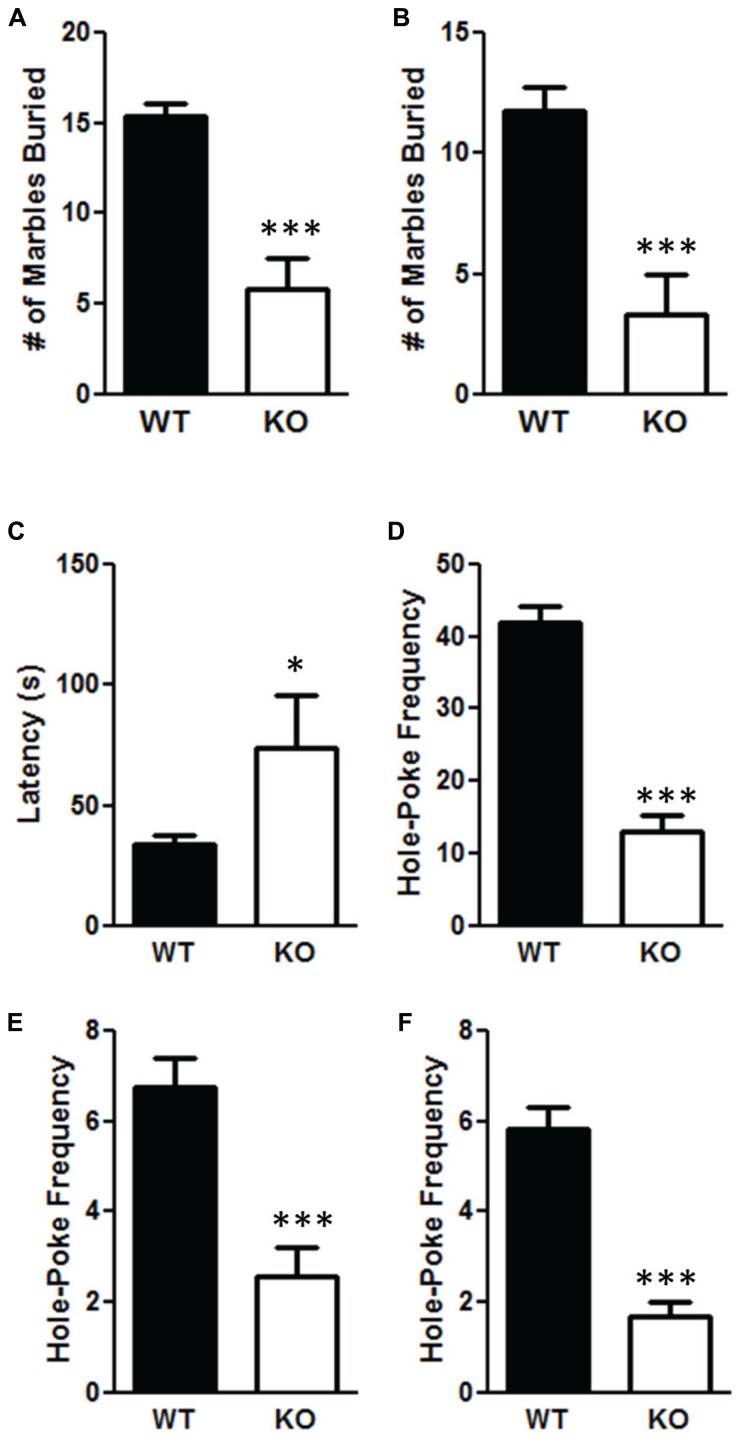
**Deletion of PTEN results in deficits in repetitive behaviors. (A)** NS-Pten knockout (KO) mice buried fewer marbles at the 75% level; **(B)** and buried fewer marbles completely. NS-Pten KO mice showed a similar pattern of results in the hole-board test. **(C)** KO Pten mice had a longer latency to the first hole poke. **(D)** KO mice also performed fewer hole-pokes in the entire board; **(E)** performed fewer hole-pokes in the center four holes of the board; **(F)** and performed fewer hole-pokes repeated in the same hole compared to WT mice. The bars represent the mean with Standard Error of the Mean. The asterisks (***, or *) indicate a significant group difference at respective *p* values (*p* < 0.001 or *p* < 0.05, respectively).

The NS-Pten KO mice have significant alterations in repetitive behaviors. The NS-Pten KO mice buried fewer marbles in a 30 min marble burying test when measuring at 75% *t*(1,43) = 5.9, *p* < 0.001 (**Figure 4A**); and 100% of a marble buried *t*(1,55) = 4.8, *p* < 0.001 (**Figure 4B**). For marble burying the sample size was 30 for WT and 15 for KO. NS-Pten KO mice also show an alteration in the hole-board nose poke activity. They have a longer latency to the first hole-poke *t*(1,50) = 2.5, *p* < 0.05 (**Figure 4C**), perform fewer hole pokes in the 10 min trial *t*(1,50) = 7.2, *p* < 0.001 (**Figure 4D**); perform fewer hole pokes in the center *t*(1,50) = 3.9, *p* < 0.001 (**Figure 4E**); and fewer repeated pokes in the same hole *t*(1,50) = 5.2, *p* < 0.001 (**Figure 4F**). For the hole board test the sample size was 36 for WT and 16 for KO.

We found no differences in the total number of vocalizations emitted from pups tested on postnatal day 10 or postnatal day 12. We analyzed the number of vocalizations emitted at different frequencies. We did not find any differences for the number of vocalizations on postnatal day 10 at 50 Hz *t*(1,49) = 0.15, *p* = 0.87; 60 Hz *t*(1,49) = 0.63, *p* = 0.53; 70 Hz *t*(1,49) = 0.1, *p* = 0.92; 80 Hz *t*(1,49) = 1.6, *p* = 0.12. The values for the frequency of vocalizations emitted for WT for 50, 60, 70, and 80 Hz were: 78.4 ± 8.8; 134.7 ± 12.5; 75.2 ± 10.23; 78.1 ± 15.8, respectively. The values for the frequency of vocalizations emitted for KO for 50, 60, 70, and 80 Hz were: 80.8 ± 14.3; 121.1 ± 18.4; 73.3 ± 17.2; 119.7 ± 20.7, respectively. We also did not find any differences for the number of vocalizations on postnatal day 12 at 50 Hz *t*(1,45) = 0.10, *p* = 0.92; 60 Hz *t*(1,45) = 1.2, *p* = 0.20; 70 Hz *t*(1,45) = 0.81, *p* = 0.42; 80 Hz *t*(1,45) = 0.15, *p* = 0.88. The values for the frequency of vocalizations emitted for WT on postnatal day 12 for 50, 60, 70, and 80 Hz were: 52.1 ± 7.7; 94.9 ± 13.8; 99.5 ± 16.9; 125.0 ± 19.4, respectively. The values for the frequency of vocalizations emitted for KO for 50, 60, 70, and 80 Hz were: 50.3 ± 11.9; 51.3 ± 19.47; 64.1 ± 36.7; 117.7 ± 42.4, respectively.

### PTEN DELETION RESULTS IN HYPERACTIVITY IN THE OPEN FIELD TEST AND DECREASES ANXIETY IN ELEVATED PLUS MAZE

**FIGURE 5:**
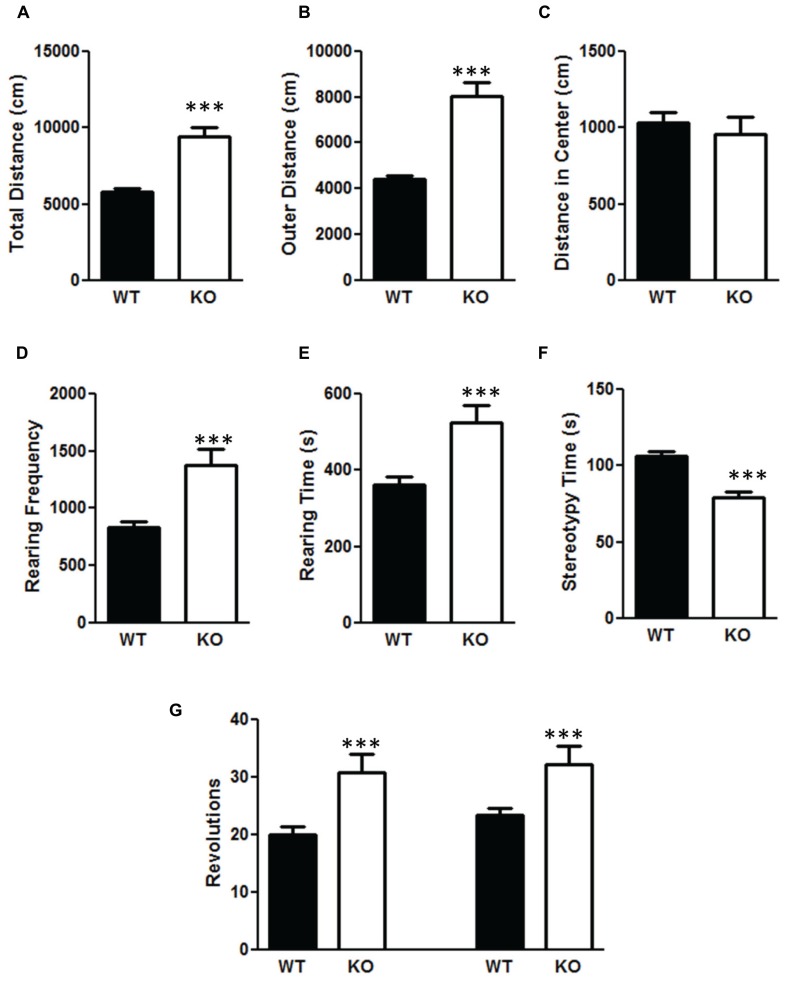
**Deletion of PTEN produces alteration in activity levels in the open field test.** Wildtype mice and NS-Pten knockout (KO) mice were hyperactive in **(A)** total distance traveled in the open field test, **(B)** were more active in the outer half of the open field test, but were no different in **(C)** center distance. **(D)** The KO mice reared more than the WT mice and **(E)** and spent more time rearing. **(F)** However, they spent less time engaging in stereotypy behavior. **(G)** The KO mice also performed more clockwise (left graphs) and more counter-clockwise revolutions (right bar graph) than the WT mice. The bars represent the mean with Standard Error of the Mean. The asterisks (***) indicate a significant group difference at *p* < 0.001.

There was a significant increase in total distance moved in the NS-Pten KO mice compared to WT mice *t*(1,53) = 6.84, *p* < 0.001 (**Figure 5A**). The increase was mostly due to movement in the perimeter. There was a significant increase in outer area/perimeter distance *t*(1,53) = 7.9, *p* < 0.001 (**Figure 5B**), but no difference between groups in the inner distance *t*(1,53) = 0.55, *p* = 0.58 (**Figure 5C**). We also measured rearing behavior in the open field through the automated detection equipment. There was a significant increase in rearing frequency *t*(1,53) = 4.6, *p* < 0.0001 (**Figure 5D**) and an increase in total time rearing *t*(1,53) = 3.6, *p* < 0.001 (**Figure 5E**) in the NS-Pten KO mice compared to WT mice. There was a similar increase in the number of clockwise rotations *t*(1,53) = 3.8, *p* < 0.001 (**Figure 5G**, left graph), and counterclockwise rotations *t*(1,53) = 3.1, *p* < 0.001 (**Figure 5G**, right graph) in NS-Pten KO mice compared to WT mice. However, there was not an increase in all behaviors. There was a significant decrease in stereotypy time *t*(1,53) = 4.8, *p* < 0.001 (**Figure 5F**) in NS-Pten KO mice compared to WT mice. The stereotypy count represents self-grooming in the mice. The sample size for the open field test was 40 for WT and 15 for KO.

**FIGURE 6:**
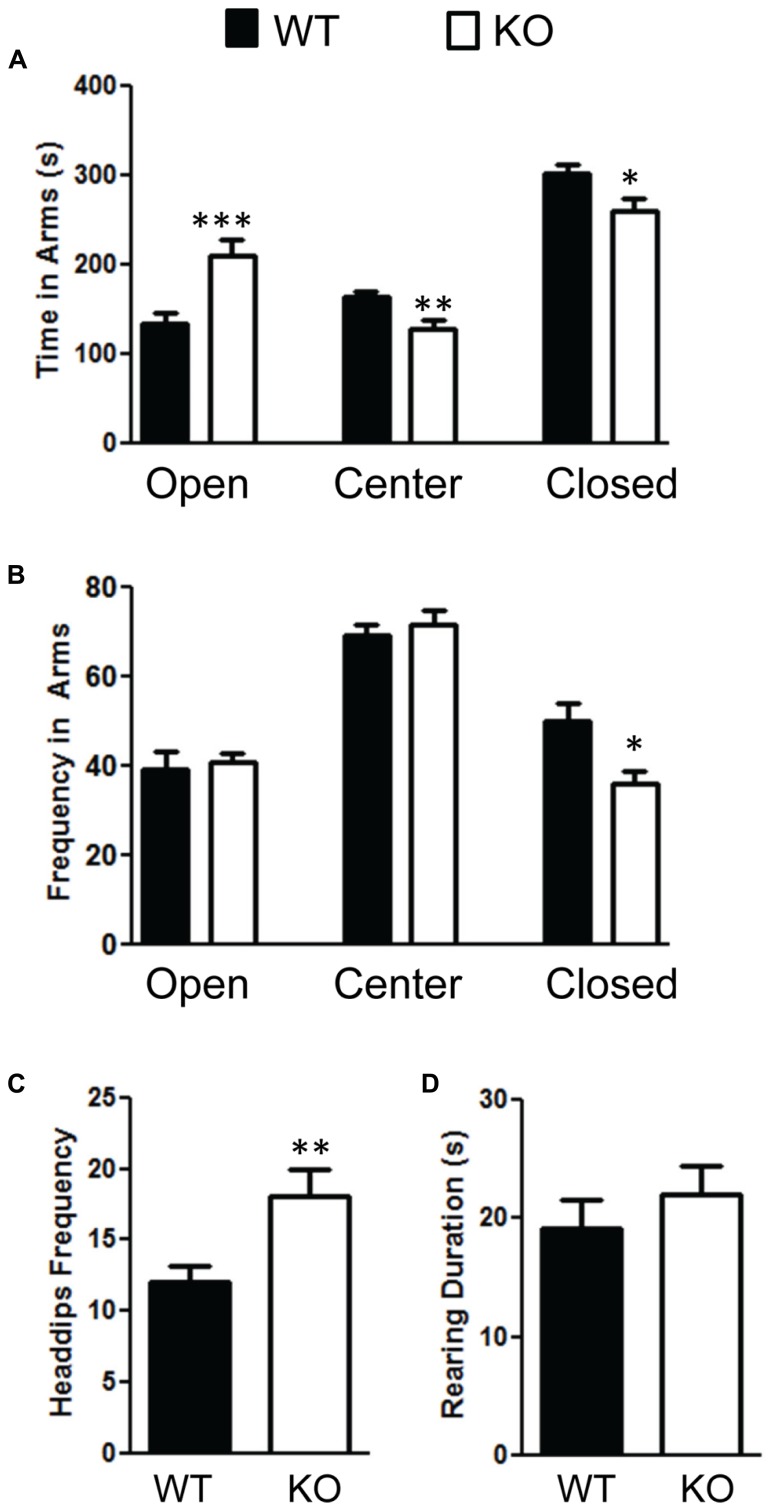
**Deletion of PTEN alters anxiety in the elevated plus maze test. (A)** NS Pten knockout (KO) mice spent more time in the open arm and less time in the center and closed arms. **(B)** NS-Pten KO mice visited the open and center areas the same but visited the closed arms less frequently. Furthermore, **(C)** NS-Pten KO mice performed more head dips in the open arm, but were not different than wildtype (WT) in the amount of rearing in the closed arms **(D)**. The bars represent the mean with Standard Error of the Mean. The asterisks (***, **, or *) indicate a significant group difference at respective *p* values (*p* < 0.001; *p* < 0.01; *p* < 0.05, respectively).

We also measured anxiety through the elevated plus-maze test and found that the NS-Pten KO mice were less anxious. NS-Pten KO mice spent more time in the open arms *t*(1,37) = 3.7, *p* < 0.001; less time in the center field *t*(1,37) = 3.1, *p* < 0.01; and less time in the closed arms *t*(1,37) = 2.5, *p* < 0.05 compared to WT mice (**Figure 6A**). We then analyzed their movement in each arm. We found no difference in the number of times the NS-Pten KO mice entered the open arms *t*(1,37) = 0.28, *p* = 0.78; center arm *t*(1,37) = 0.66, *p* = 0.51; but did find a decrease in the number of entries in the closed arms *t*(1,37) = 2.7, *p* < 0.05 compared to WT mice (**Figure 6B**). We then investigated the number of head dips and rearing in the elevated plus maze. We found an increase in the number of head dips performed by the NS-Pten KO mice compared to WT mice *t*(1,37) = 2.9, *p* < 0.01 (**Figure 6C**). We did not find a difference in the number of rearing events in the closed arms *t*(1,37) = 0.63, *p* = 0.53 (**Figure 6D**). We did not observe rearing behavior in either group in the open arms (data not shown). The sample size for the plus maze test was 24 for WT and 16 for KO.

### PTEN DELETION INCREASES PI3K/AKT/mTOR SIGNALING

**FIGURE 7:**
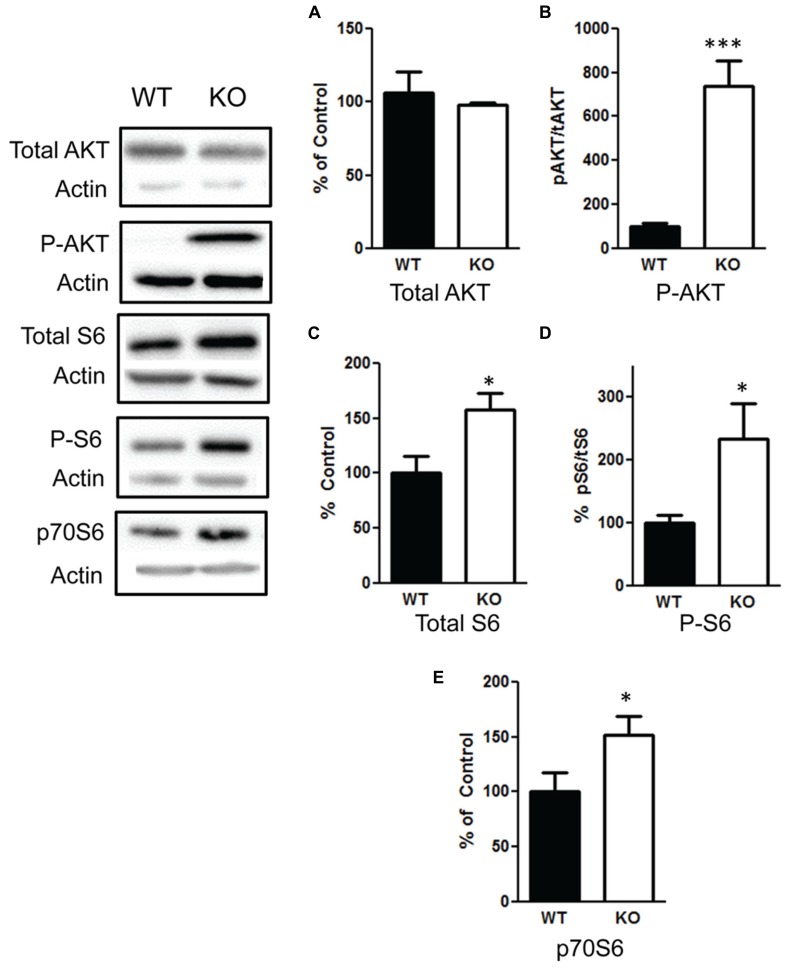
**Deletion of PTEN results in hyperactivation of the PI3K/AKT/mTOR pathway. (A)** Pten-knockout (KO) mice show similar levels of total AKT levels compared to wildtype (WT) mice. **(B)** KO mice show elevated levels in the ratio of phosphorylated AKT over total AKT levels compared to wildtype (WT) mice. **(C)** KO mice show elevated levels of total S6 and **(D)** an increase in the ratio of phosphorylated S6 over total S6 levels compared to wildtype (WT) mice. **(E)** There was also an increase in S6K in KO mice compared to WT mice in the hippocampus. The bars represent the mean with Standard Error of the Mean. The asterisks (*** or *) indicate a significant group difference at respective *p* values (*p* < 0.001; *p* < 0.05, respectively).

We confirmed that deletion of PTEN in the hippocampus results in hyperactivation of the PI3K/AKT/mTOR pathway by measuring changes in AKT and mTOR. We found no difference in total AKT levels *t*(1,10) = 0.59, *p* = 0.57 (**Figure 7A**), *n* = 6 per group. However, there was a significant increase in the ratio of phosphorylated AKT/total AKT *t*(1,10) = 5.5, *p* < 0.001 (**Figure 7B**), *n* = 6 per group. We observed an increase in total S6 *t*(1,10) = 2.7, *p* < 0.05 (**Figure 7C**), *n* = 6; the ratio of phosphorylated S6/total S6 in the NS-Pten KO mice compared to the WT mice *t*(1,10) = 2.5, *p* < 0.05 (**Figure 7D**), *n* = 6. We also found a significant increase in p70S6 (S6K) protein *t*(1,28) = 2.1, *p* < 0.05, *n* = 15 in the hippocampus of NS-Pten KO mice (**Figure 7E**).

### PTEN DELETION DECREASES mGlurR, INCREASES FMRP, AND ALTERS SEVERAL SYNAPTIC SCAFFOLDING PROTEINS

**FIGURE 8:**
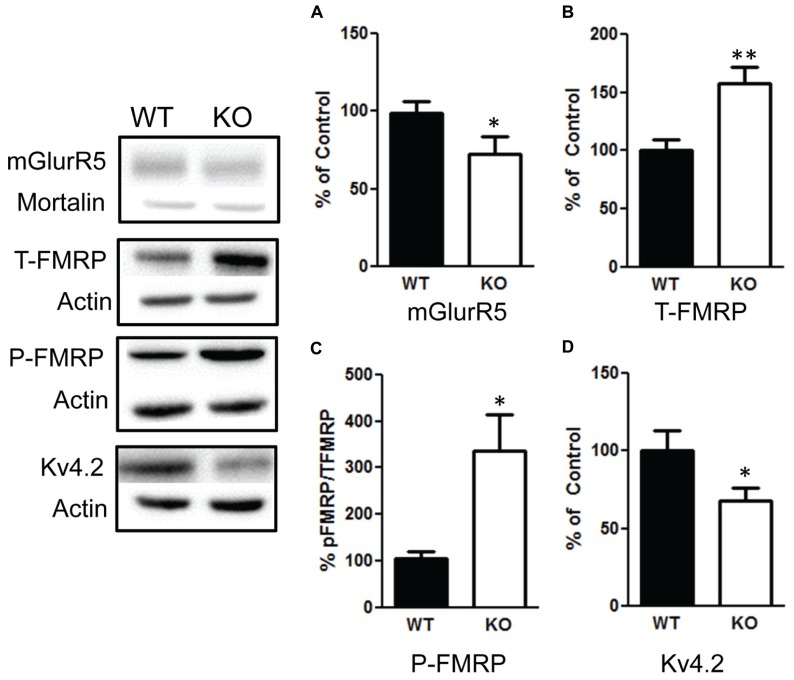
**Deletion of PTEN alters mGluR5-FMRP signaling. (A)** Knockout (KO) mice show a significant decrease in mGluR compared to wildtype (WT) mice. **(B)** KO mice show elevated total levels in fragile X mental retardation protein (FMRP) compared to WT mice and **(C)** an increase in the ratio of phosphorylated FMRP over total FMRP levels compared to WT. **(D)** KO mice had a significant decrease in Kv4.2 channels compared to WT. The bars represent the mean with Standard Error of the Mean. The asterisks (** or *) indicate a significant group difference at respective *p* values (*p* < 0.01; *p* < 0.05, respectively).

We then examined the influence of the deletion of PTEN on the mGlurR-FMRP signaling. We found a significant increase in total FMRP levels in the NS-Pten KO mice *t*(1,10) = 3.2, *p* < 0.01 (**Figure 8B**), *n* = 6. There was also a significant increase in the ratio of phosphorylated FMRP over total FMRP *t*(1,10) = 2.9, *p* < 0.05 (**Figure 8C**), *n* = 6. This increase in total and phosphorylated FMRP is not in response to an increase in group 1 metabotropic glutamate receptor (mGluR) since NS-Pten KO mice had a significant decrease in mGluR *t*(1,32) = 2.9, *p* < 0.05 (**Figure 8A**), *n* = 17. Previous studies have found that FMRP can directly alter the level of Kv4.2 levels (Gross et al., 2011; Lee et al., 2011). Indeed, we found a significant decrease in Kv4.2 channels in KO mice *t*(1,22) = 2.1, *p* < 0.05 (**Figure 8D**), *n* = 12.

**FIGURE 9:**
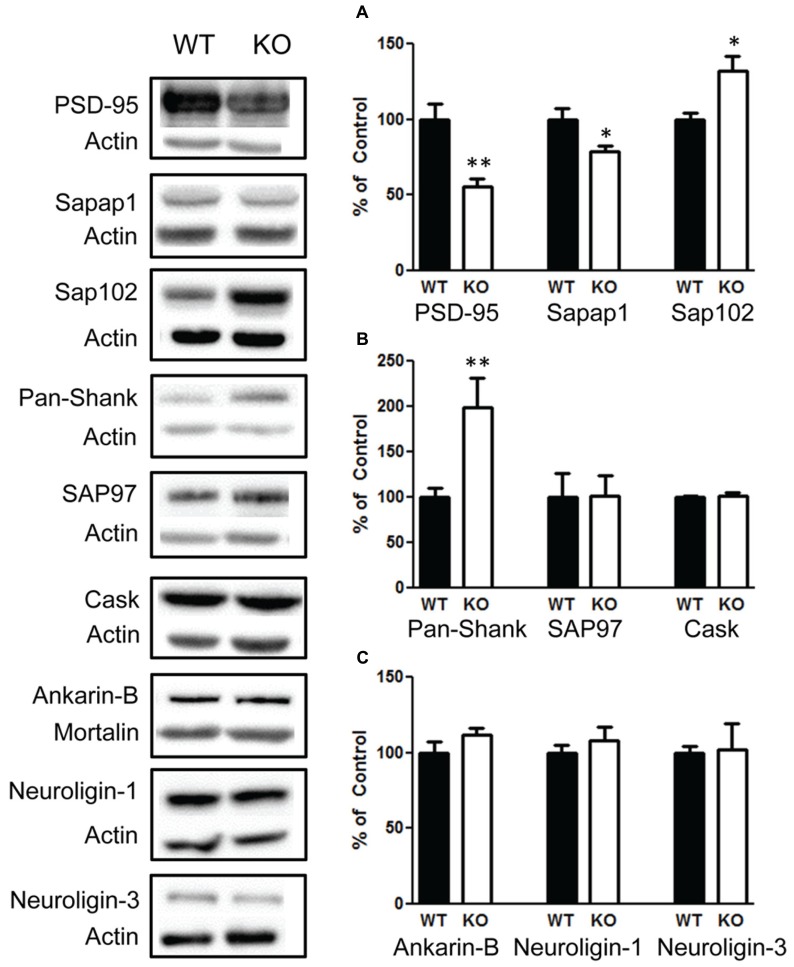
**Deletion of PTEN alters several synaptic scaffolding proteins in the hippocampus. (A)** Knockout (KO) mice show a significant decrease in PSD-95 (left figure) and in sapap1 compared to wildtype (WT) mice (center figure), and KO mice show elevated sap102. **(B)** Pan-shank levels compared to WT mice (left graph); KO and WT mice show no differences in the amount of the scaffolding proteins SAP97 (center graph); CASK (right graph). **(C)** There were also no differences in the amount of Ankyrin-B (left graph), neuroligin-1 (center graph) or neuroligin-3 protein compared to WT mice (right graph). The bars represent the mean with Standard Error of the Mean. The asterisks (** or *) indicate a significant group difference at respective *p* values (*p* < 0.01; *p* < 0.05, respectively).

Due to the role of FMRP in regulating synaptic proteins, we investigated a number of synaptic proteins to determine whether they are altered in response to changes in the PI3K/AKT/mTOR pathway and FMRP. We found that there was significant decrease in PSD-95 *t*(1,10) = 3.9, *p* < 0.01 (**Figure 9A**, left graph), *n* = 6; and sapap1 *t*(1,10) = 2.5, *p* < 0.05 (**Figure 9A**, center graph), *n* = 6. However, we also found a significant increase in sap102 *t*(1,10) = 2.8, *p* < 0.05 (**Figure 9A**, right graph), *n* = 6; and an increase in Pan-Shank *t*(1,22) = 2.96, *p* < 0.01 (**Figure 9B**, left graph), *n* = 12; but no change in SAP97 *t*(1,10) = 0.05, *p* = 0.95 (**Figure 9B**, center graph), *n* = 6. We also investigated whether presynaptic glutamatergic proteins may be altered. We found no changes in CASK *t*(1,10) = 0.58, *p* < 0.57 (**Figure 9C**, right graph), *n* = 6; or in Ankyrin-B *t*(1,10) = 1.3, *p* = 0.20 (**Figure 9C**, left graph), *n* = 6. In addition, there were no differences in Neuroligin-1 *t*(1,10) = 0.7, *p* = 0.49 (**Figure 9C**, center graph), *n* = 6; and Neuroligin-3 *t*(1,10) = 0.1, *p* = 0.91 (**Figure 9C**, right graph).

## DISCUSSION

There were two main objectives for the experiments in this study. One was to determine the autism-like behavioral deficits in mice with deletion of PTEN and the second objective was to determine the synaptic alterations in the KO mice and the possible mediator for these alterations. We found that deletion of PTEN results in social behavior deficits, repetitive behavior deficits, but did not result in deficits in pup emitted UVs. We also found that deletion of PTEN results in several alterations in synaptic scaffolding proteins, and results in a decrease in group 1 mGluR and an increase in fragile X mental retardation protein (FMRP).

Our study has expanded on a previous study that reported social behavior deficits in mice with deletion of PTEN (Kwon et al., 2006). In this study they used a social interaction test and found that Pten-KO mice engage in less social interaction behavior with a novel conspecific juvenile. We further expanded on the social behavior measures by including the three chamber social behavior test and the social partition test since the cre conditional KO we used in our study had not been characterized. In the Kwon et al. (2006) study, they used a neuron-specific enolase promoter-driven cre transgenic line, which produces a deletion of PTEN in the CA3, polymorphic layer of the hippocampus, and the dentate gyrus. In our cre transgenic line, the deletion is expressed under the control of a modified glial fibrillary acidic protein (Gfap; Toggas et al., 1994). Through the use of this promoter the Cre activity in the hippocampus is localized mainly in the granule cells of the dentate gyrus (Kwon et al., 2001). Therefore, our report is the first to demonstrate that Gfap-cre Pten conditional KO results in social behavior deficits.

One assumption in the social chamber and social partition test is that the animal can recognize a novel animal. Mice rely heavily on olfaction to detect and differentiate other mice (Ferguson et al., 2000; Arakawa et al., 2007). It is increasingly becoming common to measure the animal’s ability to distinguish different novel odors, including social odors to determine whether the animal has anosmia. The inability to distinguish different odors could result in false positives for social impairments. We found that NS-Pten KO mice can detect different novel odors, habituate to the repeated presentation of the odors, and dishabituate when presented with another odor. We did not observe any statistical differences between the two groups across any of the odors, but we did observe a trend where the NS-Pten KO habituates to the social odor slightly more quickly than the WT mice. However, the social deficits in NS-Pten KO are not due to the inability to distinguish different odors.

We used the marble burying test and hole-board test to measure repetitive behaviors and observe deficits in both tests. To our knowledge this is the first report of repetitive behavior deficits found in mice with PTEN deletion. Even though these two tests clearly demonstrate deficits in repetitive behavior their results are contrary to the observations in the open field test. In the open field test the NS-Pten KO mice show hyperactivity, an increase in rearing, an increase in circling behavior, but a decrease in repetitive behavior. The Fusion software used to measure locomotion in the open field defines the repetitive behavior as self-grooming behavior. Therefore, marble burying, nose poke, and self-grooming all show a decrease in repetitive behavior. It is likely that the hyperactivity and increase in rearing are independent from repetitive behavior. The increase in locomotion in mice with deletion of PTEN has been reported previously (Kwon et al., 2006). The open field test is often used as a measure of motor ability and not as a measure of repetitive behavior (Crawley, 1999). Therefore, the NS-Pten KO mice show deficits in repetitive behavior and separate alterations in motor behavior. Additional studies would be needed to address the motor behavior deficits in these mice.

We did not find any alterations in the communication aspect of ASD since no differences were observed in UVs in pups on postnatal day 10 or 12. We measured vocalizations on day 10 and 12 since the deletion of PTEN in the Gfap-cre KO is present by postnatal day 5, and we wanted to measure the vocalizations at a time when deletion of the gene would manifest. However, it is possible that the impact of the gene deletion is not immediate. PTEN deletion causes a progressive increase in brain size and cellular dysfunction (Kwon et al., 2001; Takeuchi et al., 2013). Future studies could examine UVs in adult mice to investigate the influence of the cumulative impact of PTEN deletion on mouse communication.

We first confirmed that NS-Pten KO mice have hyperactivation of the PI3K/AKT/mTOR pathway. We observed no change in total AKT, but found a significant increase in the phosphorylated AKT over total AKT in the hippocampus. We found an increase in total S6 and an increase in the ratio of phosphorylated S6 over total S6 in the hippocampus. We further confirmed this activation by finding an increase in S6K in the hippocampus. There have been several reports of aberrant mTOR signaling in mouse models of fragile X mental retardation (Narayanan et al., 2008; Sharma et al., 2010; Bhattacharya et al., 2012). There is an increase in FMRP and a decrease in mGluR in the hippocampus of NS-Pten KO mice. FMRP is believed to inhibit translation of synaptic proteins (Bassell and Warren, 2008). In particular, FMRP binds to the mRNAs of several ion channel proteins (Bell et al., 1991; Darnell et al., 2011). One ion channel that has been shown to be altered in mouse models of fragile X is the potassium ion channel Kv4.2 (Gross et al., 2011; Lee et al., 2011; Routh et al., 2013). We examined this protein and found a significant decrease in Kv4.2 in the hippocampus of NS-Pten KO mice. Kv4.2 channels mediate the A-type K^+^ channel and are essential for normal dendritic excitability and function (Hoffman et al., 1997; Ramakers and Storm, 2002).

In addition to Kv4.2, we examined the impact of PTEN deletion on a number of synaptic scaffolding proteins. We found a decrease in PSD-95 and sapap1 but an increase in Pan-Shank and sap102. We did not observe a changes in CASK, which a protein that belongs to the family of membrane associated guanylate kinase (MAGUK) proteins (Hsueh, 2006). We also did not observe a change in Ankyrin-B or any changes in Neuroligin-1 or Neuroligin-3. We examined these proteins since many have been implicated in autism (Ey et al., 2011). The decrease in PSD-95 is of significance since it is a key component at excitatory synapses that mediates the neurexin/neuroligin/Shank pathway. This pathway is believed to play a major role in autism. Mice with deletion of the gene for PSD-95 exhibit altered repetitive behaviors, abnormal communication, and social behaviors (Feyder et al., 2010). It is not surprising that PSD-95 was decreased since PSD-95 mRNAs in the dendrites have been shown to be directly associated with FMRP (Muddashetty et al., 2007; Zalfa et al., 2007).

If the alterations in Kv4.2 and the other synaptic scaffolding proteins are due to aberrant hyperactivation of FMRP then future studies could determine whether mGluR agonists can reverse some of the behavioral and molecular changes observed in NS-Pten KO mice. Mouse models of fragile X mice have provided support for the idea that excess protein synthesis is responsible for the behavioral deficits and synaptic alterations (Ronesi and Huber, 2008). Indeed, the mGluR theory of fragile X syndrome is that the absence of FMRP leads to an increase in protein synthesis (Bear et al., 2004). This theory has led to the development of the mGluR5 inhibitor CTEP, which has been found to reverse many of the fragile X symptoms in mice (Michalon et al., 2012). In contrast mice with deletion of PTEN have excessive FMRP and phosphorylated FMRP. Therefore, future studies could determine whether agonists of mGluR can reverse some of the behavioral and molecular alterations. These studies would provide insights into the relationship between PTEN and FMRP and could lead to a possible therapeutic approach for individuals with deletion of PTEN or for those with aberrant PI3K/AKT/mTOR activation.

There is increasing evidence that neurodevelopmental disorders such as ASDs, fragile X syndrome, tuberous sclerosis complex, and others are linked to synaptic abnormalities. There is also increasing evidence that there are convergent signaling pathways that may mediate these changes. We present evidence here that deletion of PTEN in a subset of neurons in the hippocampus can increase in the PI3K/AKT/mTOR pathway and alter mGluR-FMRP. These changes result in several synaptic abnormalities that have been shown to be involved in autism. Additional studies could determine whether inhibitors of mTOR or agonists of mGluR could be future therapeutics for those with neurodevelopmental disorders.

## AUTHOR CONTRIBUTIONS

Gregory D. Smith and Jessika White conducted the western blotting and analysis for the research Erin P. Arbuckle and Maribel C. Gomez conducted the odor discrimination test and scored some of the social behavior tests. Crina M. Floruta scored some of the social behavior tests. Nowrin Ahmed scored the hole board test. Gregory D. Smith conducted and scored the marble burying, elevated plus maze, and open field tests. Andrew J. Holley and Obi Okonkwo conducted the ultrasonic vocalizations research. Joaquin N. Lugo supervised the project, analyzed the data, and wrote the manuscript. All individuals contributed to the design of each test in the manuscript, assisted with the drafting of the manuscript, approved the final version of the manuscript, and have agreed to be accountable for all work to be published.

## Conflict of Interest Statement

The authors declare that the research was conducted in the absence of any commercial or financial relationships that could be construed as a potential conflict of interest.
